# Effects of cold-treatment and strain-rate on mechanical properties of NbTi/Cu superconducting composite wires

**DOI:** 10.1186/s40064-015-0860-7

**Published:** 2015-02-13

**Authors:** Mingzhi Guan, Xingzhe Wang, Youhe Zhou

**Affiliations:** Key Laboratory of Mechanics on Environment and Disaster in Western China, The Ministry of Education of China, College of Civil Engineering and Mechanics, Lanzhou University, Lanzhou, 730000 P.R. China; Institute of Modern Physics of Chinese Academy of Science, Lanzhou, 730000 P. R. China

**Keywords:** NbTi/Cu composite wires, Effect of cold-treatment, Strain-rate, Tensile response, Mechanical property

## Abstract

During design and winding of superconducting magnets at room temperature, a pre-tension under different rate is always applied to improve the mechanical stability of the magnets. However, an inconsistency rises for superconductors usually being sensitive to strain and oversized pre-stress which results in degradation of the superconducting composites’ critical performance at low temperature. The present study focused on the effects of the cold-treatment and strain-rate of tension deformation on mechanical properties of NbTi/Cu superconducting composite wires. The samples were immersed in a liquid nitrogen (LN_2_) cryostat for the adiabatic cold-treatment, respectively with 18-hour, 20-hour, 22-hour and 24-hour. A universal testing machine was utilized for tension tests of the NbTi/Cu superconducting composite wires at room temperature; a small-scale extensometer was used to measure strain of samples with variable strain-rate. The strength, elongation at fracture and yield strength of pre-cold-treatment NbTi/Cu composite wires were drawn. It was shown that, the mechanical properties of the superconducting wires are linearly dependent on the holding time of cold-treatment at lower tensile strain-rate, while they exhibit notable nonlinear features at higher strain-rate. The cold-treatment in advance and the strain-rate of pre-tension demonstrate remarkable influences on the mechanical property of the superconducting composite wires.

## Introduction

Superconductors have been shown to possess a variety of promising applications in electricity, medical, electronics fields, especially in particle accelerators with superconducting magnets (Allain et al. 2013). In practices, the most superconducting magnets are generally wound using composite wires containing thin superconducting (usually brittle) filaments inside a ductile metal matrix. It is well known that NbTi/Cu superconducting composites have better thermal and mechanical/electric stability at low temperature, they thus are frequently employed in various applications (Cau et al. 2010).

With the development of superconducting magnet technology, the more and larger superconducting magnets were fabricated to provide stronger/faster magnetic field (~9 T, 1.2 T/s). Although the high-temperature superconductors have been developed widely (Choi et al. 2009), the investigations on the measurement of mechanical of NbTi/Cu superconductors and magnets still were reported continuously and frequently for understanding their mechanical characteristics in a complicated environment (Zhang et al. 2011; Ghatea et al. 2014). The complicated multi-fields including the extreme high magnetic field unavoidably cause the deformation and multi-behaviors of NbTi/Cu superconducting magnets, which even lead the interruption of the excited superconducting magnet, i.e., the quenching. Wright et al. (Wright et al. 1984) conducted tensile stress experiments at temperature of 293 K, 77 K, and 4.2 K on the pure superconducting NbTi fibers, which were chemically extracted from the commercial superconducting composite wires. Their observations showed that, as the temperature decreases the fracture stress and elongation of the superconducting NbTi fibers increase. Read and Lebetter (Read and HM Ledbetter 1978) further reported elastic properties of the NbTi/Cu superconductors at 300 K and 4 K using an ultrasonic pulse-superposition technology, and some differences of elastic behavior between 300 K and 4 K were argued in their work. Focusing on the mechanical behavior depending on the low-temperature, the authors (Guan et al. 2012) recently measured the effects of variable cryogenic temperature on the tensile response for the commercial superconducting NbTi/Cu composites by using a variable temperature cryostat system and low-temperature extensometer. To achieve good mechanical performance of superconducting materials, some of researches have been involved measuring the effect of physical treatment on mechanical properties to evaluate and strengthen their service life. For instances, many commercial superconducting composites can be improved mechanically by optimizing their reinforcing materials (Vasanthamohan and Singh 1997; Al-Mosawi et al. 2006; Banno et al. 2006). However, the reinforcing materials for mechanical relief in superconductors can consume valuable space in a cooled volume. There are also a few works to study and improve the mechanical properties by pre-treatment for structural reliability in superconductivity applications. Fujimoto et al. (Fujimoto & Murakami 2012) attempted to improve mechanical properties of an innovative Gd123/Ag superconductor by adjusting samples’ void density. The results showed that the mechanical properties of the densified Gd123 bulk with low void density are better than those of the standard Gd123 bulk with voids at 77 K. The relations between the microstructure and the flexural strength or the fracture toughness of the densified Gd123 bulk have been captured. For high purity Tantalum single crystals, Tarkeuch et al. (Tareuchi et al. 1977) measured flow stress changes with the transition between the superconducting and the normal states, and with the change of the strain-rate. It observed that high strain-rate obviously dependents of flow stress of Tantalum in the superconducting and normal states. In the winding and insulation processes, a certain bending strain was applied to the superconducting tap and then released at room temperature, which is defined as pre-bending treatment (Awaji et al. 2003). By the way of local hardening effects with applying the bending strain, it was found the improvement of mechanical properties could be obtained for CuNb/(Nb,Ti)_3_Sn superconducting coils made by a react and wind (R&W) method.

In the present study, we carried out experiments on the practical NbTi/Cu superconducting composite wires under thermo-mechanical field. The major tensile mechanical parameters of the pre-treated NbTi/Cu composite wire, such as ultimate tensile strength and elongation to fracture, yield strength, were measured at cryogenic temperature in the strain rate range of 10^−4^ ~ 10^−2^ s^−1^ and room temperature. It was demonstrated the nonlinear features for the multi-field effect of cold-treatment holding time and strain-rate on the NbTi/Cu composite wires’ mechanical behaviors.

## Experimental detail

The specimens cut from commercial multifilamentary superconducting composite wires consisting of niobium–titanium filaments in a copper matrix (NbTi/Cu) were used in our experiment. The Cu/NbTi volume ratio of the specimens is 4.33, and the composite wire contains 36 filaments with a twist pitch of 50 mm (Guan et al. 2012). The wires have been used currently in lots of superconducting coils in practical applications.

**Figure 1:**
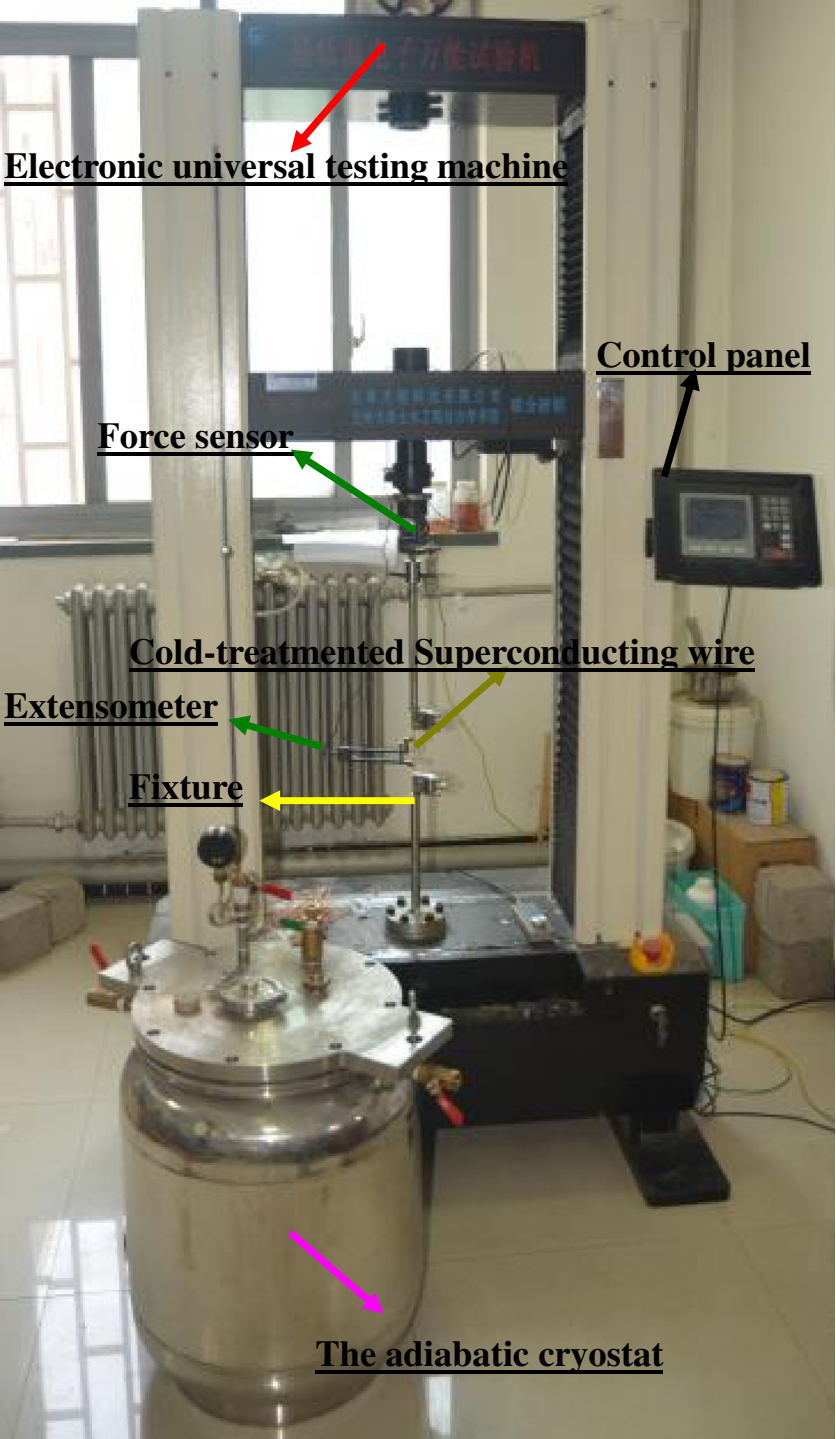
**Experiment set-up: adiabatic cryostat system and mechanical loading apparatus.**

In our tests, we paid simultaneously attentions to the effects of the cold-treatment and strain-rate of mechanical properties of NbTi/Cu superconducting samples under tension deformation. Before the tensile test, all samples were immersed in liquid nitrogen (LN_2_) cryogenic chamber with double vacuum layers (see Figure 1), for the pre-cold-treatment with 18-hour, 20-hour, 22-hour and 24-hour, respectively. And the mechanical loading to the pre-cold-treated samples were implemented by a standard electronic universal testing machine at room temperature. The uncertainty of displacement and load measurement on the testing machine are 0.001 mm and 3 N, respectively. To perform the tension tests with different strain rates, the crosshead speeds of the apparatus were set at 0.5 mm/min, 5 mm/min, 50 mm/min and 100 mm/min. A small cryogenic-type extensometer (Epsilon 3542) was used to measure the strains of the specimens, which can be performed with high stability and precision in a large temperature range from room temperature to 4.2 K. The calibration of extensometer was done at cryogenic temperature by Epsilon Company Limited (U.S.). The weight of the extensometer is less than 200 g, there may arise non neglectable force on the side of the wire caused by the weight of extensometer, as shown in Figure 1. To eliminate this side force, one way is to hang the extensometer for balancing its weight by a elastic rope, and the other way is to apply a small tension preloading to the wire before tests. The experiment measurement illustrated that the ways is reasonably accepted.

According to the definition of nominal strain rate (Tareuchi et al. 1977), the corresponding nominal strain rates were evaluated respectively as 1.67 × 10^−4^ s^−1^, 1.67 × 10^−3^ s^−1^, 1.67 × 10^−2^ s^−1^ and 3.33 × 10^−2^ s^−1^. The errors in the reported wires’ mechanical parameters at high strain rate usually arise from accuracy errors of the extensometer, force sensor, and grips. In our tests, the extensometer errors are estimated to be about ± 1 × 10^−6^ s^−1^; force sensor errors are estimated to be about ± 1 × 10^−5^ s^−1^. To achieve quite good recording data, at least six samples were used for each tension measurement and the averaged observation results were reported here.

## Results and discussion

**Figure 2:**
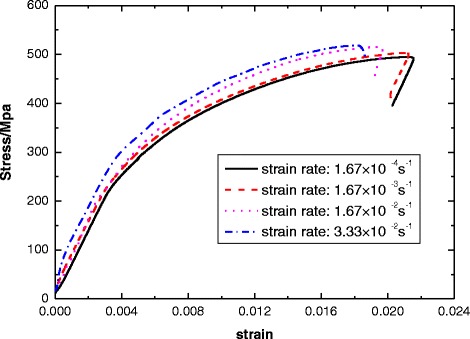
**The stress–strain curve of pre-cold-treatment NbTi/Cu composite wire with 24H holding-time at different strain rates.**

**Figure 3:**
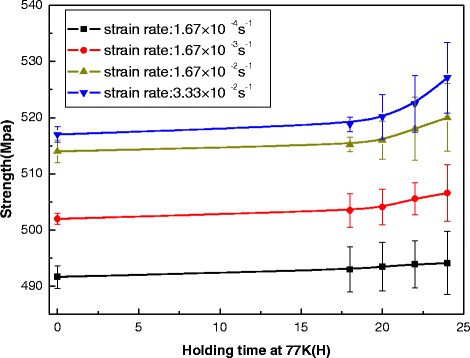
**Ultimate tensile strengths of the NbTi/Cu composite wire dependence upon the holding-time for different strain rates.**

**Figure 4:**
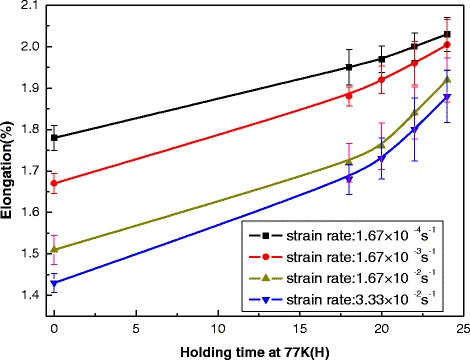
**Elongation of the NbTi/Cu composite wire dependence upon the holding-time for different strain rates.**

**Figure 5:**
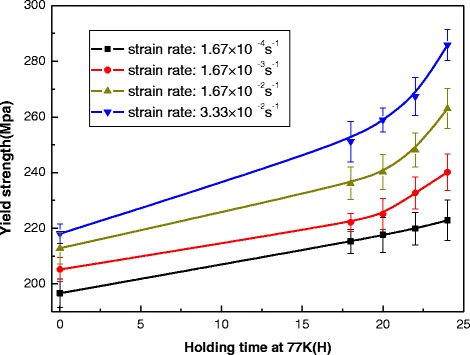
**Yield strength of the NbTi/Cu composite wire dependence upon the holding-time for different strain rates.**

The nominal stress–strain curves of pre-cold-treatment NbTi/Cu composite wires were tested at different strain rates during the tests. Figure 2 shows the stress–strain curve of pre-cold-treatment NbTi/Cu composite wire with 24H holding-time at different strain rates. Then, some primary mechanical properties, including the strength, elongation at fracture and yield strength, were extracted and reported. Based on these data generated on the superconducting samples with the diverse holding time of pre-cold-treatment and strain rate, we can draw the conclusion that the nonlinear features for the multi-field effect on the NbTi/Cu composite wires’ mechanical behaviors are indeed in existence, provided that a high strain rate condition can be achieved. The corresponding results are presented in Figures 3, 4 and 5.

Ultimate tensile strengths (UTS), which are independent of the shape and size of the superconducting samples, were investigated firstly. Under the different strain rates, Figure 3 plots the pre-cold-treatment NbTi/Cu composite wires’ UTS depending upon holding-time at 77 K. It is shown that the UTS increase with an increasing holding time, and an obvious improvement on the strength was exhibited when the holding time of pre-cold-treatment is above 20 hours. At low tensile strain-rate, i.e. 1.67 × 10^−4^, the UTS of the superconducting samples with pre-cold-treatment is about 0.6% higher than the one without pre-cold-treatment of 24 h holding-time at 77 K. However, the value can be enhanced to about 2% at high strain-rate of 3.33 × 10^−2^ s^−1^. The long periods of time of pre-cold-treatment (more than 1 day) will help for strengthening wires’ UTS. It is also shown that the samples’ ultimate tensile strength at higher strain rate is always larger than that at lower strain rate for different holding-time. It is illustrated that UTS of NbTi/Cu composites can be enhanced by improving tensile strain-rate. Consequently, during winding of superconducting coils at room temperature, a winding pre-tension under higher rate for pre-cold-treatment NbTi/Cu composite wires, may be applied to improve the mechanical stability of the coils. Furthermore, at higher strain-rate, the nonlinear relations between UTS and holding-time of pre-cold-treatment are more notable.

Elongation at break, also known as fracture strain, is the ratio between changed length and initial length after breakage of the test specimen. It always represents the capability of a material to resist changes of shape without crack formation. The elongation of superconducting wires plays an important role in the winding process. Wires with smaller elongation may lead easily to the motion of superconductors during the fast excitation, and hence the elongation at fracture is usually the principal mechanical concern. The elongation at break of the NbTi/Cu composite is shown in Figure 4. It is shown that the elongations nonlinearly increase withholding-time at 77 K from high strain-rate to low strain-rate. At $\dot{\varepsilon}=1.67\times {10}^{\hbox{-} 4}{\mathrm{s}}^{\hbox{-} 1},$ the elongations of the superconducting samples with pre-cold-treatment of 24 h holding-time at 77 K is about 15% higher than the ones without pre-cold-treatment. When the tensile strain-rate is increased to 3.33 × 10^−2^ s^−1^, a larger value of improvement on elongation of samples is obtained about 30%. In addition, one also can find that, from Figure 3, the elongation of stretched superconducting samples at low strain-rate is always higher than the one at high strain-rate for different holding-time. And the large elongation will be requested for the manufacturing of some special-shaped and pulsed superconducting coils.

A yield strength is defined in engineering and materials science as the stress at which a material begins to deform plastically. Prior to the yield point, the material will deform elastically and will return to its original shape when the applied stress is removed. Once the yield point is passed, some fractions of the deformation will be permanent and non-reversible. For superconducting wires, the notable degradation of its critical current dependence on the large and irreversible strain is always the considerable factor in the application. The yield strength of NbTi/Cu composite wires varying with the holding-time and strain-rate is plotted in Figure 5. It shows that the yield strength increases linearly with the holding-time at low tensile strain rate, when the strain-rate is higher (e.g., above 10^−3^ s^−1^), the yield strength increases exponentially with the holding-time. Additionally, the yield strength of the superconducting samples without pre-cold-treatment at strain rate 3.33 × 10^−2^ s^−1^ is about 11% higher than the one at train rate 1.67 × 10^−4^ s^−1^. And for the samples with pre-cold-treatment of 24 h holding-time at 77 K, the value can be change to about 24%. However, it should be noted that the variation of yield strength mainly involves the yield behavior of copper in the superconducting composite at high strain-rate. It is because the yield strength of NbTi filaments in the composite is always zero at the temperature region from RT to LNT (Guan et al. 2012).

Finally, the Young’s modulus of the NbTi/Cu composite wires was investigated at room temperature for different holding times of pre-cold-treatment and strain rates. The Young’s modulus of the samples were evaluated steadily by stress–strain curves to be 98 ~ 105 GPa at 293 K, which is varied in narrow region almost independent on the holding-time or strain-rate. And in our tested rang (e.g., 10^−4^ s^−1^ ~ 10^−2^ s^−1^), with the strain rate increasing, it have been clarified that the Young’s modulus of NbTi/Cu composite wires as a constant one. It may be reasonable to take the Young’s modulus of NbTi/Cu composite wires as a constant one at room temperature and small strain rates.

## Conclusion

The effects of cold-treatment and strain-rate on the tensile responses of NbTi/Cu superconducting composite wires were addressed to improve their mechanical properties. The observations exhibit that the ultimate tensile strength, elongation and yield strength all depend simultaneously upon the holding time of cold-treatment and tensile strain rate. The mechanical properties of the superconducting wires are linearly dependent on the holding time of cold-treatment at lower tensile strain-rate, while notable nonlinear features were revealed at higher tensile strain-rate. The UTS and yield strength of samples with and without pre-cold-treatment at high strain-rate are both higher than the ones at low strain-rate, and the elongation at fracture of samples with and without pre-cold-treatment at high strain-rate are lower than the one at low strain-rate. On the basis of the observation results, one can further explore on increasing the filling factor of conductor in superconducting coils and improving the mechanical stability of superconducting magnet by applying proper pre-cold-treatment and tensile strain-rate during winding the superconducting composite coils.
